# Exploring HSP90α and hs-CRP using AI models to predict prognosis in advanced hepatocellular carcinoma treated with PD-1 inhibitors and targeted therapy

**DOI:** 10.3389/fphar.2025.1726967

**Published:** 2025-12-12

**Authors:** Zhen-Ying Wu, Xueting Li, Lu Yang, Yuhui Shi, Xianguo Liu, Lianbin Wen, Yanqiong Song, Wanyun Du, Yulin Tu, Qian Wei, Junqi Liu, Hongyan Li, Pan Wang

**Affiliations:** 1 Department of Medical Administration, Panzhihua Central Hospital, Panzhihua, Sichuan, China; 2 Department of Oncology, 363 Hospital, Chengdu, China; 3 Department of Oncology, Chongqing General Hospital, Chongqing University, Chongqing, China; 4 Department of Geriatric Cardiology, Sichuan Academy of Medical Sciences and Sichuan Provincial People’s Hospital, Chengdu, China; 5 Department of Radiotherapy, Sichuan Cancer Hospital and Institute, Sichuan Cancer Center, School of Medicine, University of Electronic Science and Technology of China, Chengdu, China; 6 Department of Pharmacy, Southwest Medical University, Luzhou, China; 7 Department of Ophthalmic Optics, Southwest Medical University, Luzhou, China; 8 Department of Medical Imaging, Southwest Medical University, Luzhou, China; 9 Basic Medicine College, Panzhihua University, Panzhihua, China; 10 Department of Anesthesiology, The Affiliated Traditional Chinese Medicine Hospital, Southwest Medical University, Luzhou, China; 11 Luzhou Key Laboratory of Research for Integrative on Pain and Perioperative Organ Protection, Luzhou, China; 12 Clinical Skills Center, The Affiliated Hospital, Southwest Medical University, Luzhou, China

**Keywords:** artificial intelligence, PD-1 inhibitors, hepatocellular carcinoma, Hsp90α, Hs-CRP

## Abstract

**Objective:**

This study investigates the roles of heat shock protein 90α (HSP90α) and high-sensitivity C-reactive protein (hs-CRP) in the progression and prognosis of advanced hepatocellular carcinoma (HCC) patients undergoing immunotherapy. By integrating these biomarkers with artificial intelligence (AI), we aim to elucidate the complex interactions between tumor stress, immune responses, and tumor progression.

**Methods:**

This retrospective analysis includes 644 patients with advanced HCC who received PD-1 inhibitors and targeted therapy across 3 tertiary hospitals in China from 2016 to 2023. The patients were randomly divided into training (70%) and validation (30%) sets. Independent prognostic factors for overall survival (OS) were identified using LASSO and stepwise Cox regression. Five machine learning models were built, and their performance was evaluated using Receiver Operating Characteristic (ROC) curves, Decision Curve Analysis (DCA), and calibration curves.

**Results:**

Patients with high HSP90α expression had a median OS of 7.7 months compared to 20.6 months for those with low expression (p < 0.001). Similarly, high hs-CRP levels were associated with OS of 11.6 months versus 30.8 months for low CRP (p < 0.001). LASSO and stepwise Cox regression identified age, CRP, HSP90α, Child-Pugh classification, tumor number, metastatic (M) status, and portal vein tumor thrombosis (PVTT) as independent prognostic markers. The Random Survival Forests (RSF) model achieved the highest C-index of 0.679, and in the validation set, it demonstrated AUC-ROC values of 0.803 at 6 months, 0.801 at 12 months, and 0.761 at 18 months. The RSF model demonstrated good calibration across all time points, and DCA showed consistently higher net benefit compared with “Treat All” and “Treat None” strategies. Additionally, High levels of CRP and HSP90α were also associated with advanced tumor stage and higher Child-Pugh classification.

**Conclusion:**

HSP90α and hs-CRP, plays a critical role in the prognosis of advanced HCC. Integrating these biomarkers with machine learning models enhances OS prediction accuracy, offering a personalized approach to cancer treatment.

## Introduction

Hepatocellular carcinoma (HCC) remains a major global health challenge, ranking as one of the most lethal malignancies (Ganesan and Kulik, 2023). Most patients present with advanced-stage disease, for which curative treatments are no longer feasible (Li H. et al., 2025). In recent years, systemic therapy—particularly immune checkpoint inhibitors (ICIs) and molecular targeted agents—has dramatically reshaped the therapeutic landscape of advanced HCC (Jiang et al., 2024). Combination regimens such as atezolizumab plus bevacizumab or camrelizumab plus apatinib have become standard first-line options, markedly improving overall survival (OS) (Finn et al., 2020; Xia et al., 2022). Nevertheless, responses to immunotherapy remain heterogeneous, and reliable biomarkers to predict clinical outcomes are still lacking.

Increasing evidence suggests that tumor stress is not a passive factor but an active regulator of tumor immunity. Chronic stress, sympathetic nervous activation, and stress-induced inflammation can modulate systemic immune responses, shaping both tumor progression and therapeutic efficacy (Oura et al., 2021; Li W. et al., 2025). Circulating biomarkers such as heat shock protein 90α (HSP90α) and high-sensitivity C-reactive protein (hs-CRP) provide an accessible window into this stress–inflammation axis (Su et al., 2022; Zhou et al., 2019; Iida et al., 2022). HSP90α, a stress-inducible molecular chaperone, stabilizes multiple oncogenic and immune checkpoint–related proteins, while hs-CRP, an IL-6–driven acute-phase reactant, reflects systemic inflammatory activity under neuroendocrine influence (Liu et al., 2015).

Understanding how tumor stress interacts with immune modulation could help refine treatment decisions in advanced HCC. However, traditional statistical models often fail to capture the complex, nonlinear interactions between stress, inflammation, and tumor immunity (Su et al., 2025). To address this limitation, the present study integrates artificial intelligence (AI) to evaluate the predictive and prognostic value of HSP90α and hs-CRP in patients receiving immunotherapy combined with targeted therapy (Wang et al., 2025; Xu J. et al., 2025).

By integrating clinical variables and biological markers through AI, this study aims to construct a robust prognostic model to predict the outcomes of advanced hepatocellular carcinoma treated with immunotherapy.

## Methods

### Patients and study design

This retrospective analysis includes 644 patients with advanced HCC who received PD-1 inhibitors and targeted therapy across 3 tertiary hospitals in China from 2016 to 2023.

Inclusion criteria were as follows:

1. Pathologically or radiologically confirmed diagnosis of advanced or unresectable HCC according to the American Association for the Study of Liver Diseases (AASLD) criteria;
2. Child–Pugh class A or B liver function;
3. Available baseline serum levels of HSP90α and hs-CRP measured approximately 1 month prior to treatment initiation.

Exclusion criteria included:

1. Concurrent diagnosis of other malignant tumors;
2. Previous liver transplantation or concurrent radiotherapy during systemic therapy;
3. Severe infection, autoimmune disease, or uncontrolled cardiovascular disease;
4. Incomplete clinical or laboratory data;
5. Loss to follow-up within 3 months after treatment initiation.

All procedures were performed in accordance with the ethical standards of the Declaration of Helsinki. The study protocol was reviewed and approved by the Ethics Committee of the Affiliated Hospital of Southwest Medical University (KY2025340). Written informed consent was obtained from all participants before treatment.

### Treatment

All patients received combination therapy with PD-1 inhibitors plus targeted therapy, which was continued until radiographic disease progression, unacceptable toxicity, or death.

OS was the primary endpoint of this study and was defined as the time interval from treatment initiation to death from any cause or last follow-up.

### HSP90α and hs-CRP

We first categorized patients into two groups based on the optimal cutoff points of HSP90α and hs-CRP expression levels. The survival differences between the high and low expression groups of HSP90α and hs-CRP were compared using Kaplan-Meier analysis and the log-rank test.

We then performed LASSO (Least Absolute Shrinkage and Selection Operator) regression to identify factors associated with OS in patients with advanced HCC, incorporating clinically relevant variables including Age, Sex, hepatitis B virus (HBV) status, white blood cell count (WBC), Neutrophil count (NEU), Alpha-Fetoprotein (AFP), Child–Pugh class, albumin–bilirubin (ALBI) grade, tumor number, tumor size, Portal vein tumor thrombosis (PVTT), T, N status, M status, and Barcelona Clinic Liver Cancer (BCLC) stage. This was followed by stepwise Cox regression to refine the model and determine independent prognostic factors. Variables with significant association in univariate analyses were entered into the multivariate Cox model, with backward stepwise selection to identify the most robust predictors of OS.

### AI model construction

All patients were randomly divided into a training set and a validation set in a 7:3 ratio. In the training set, the final identified independent prognostic factors were incorporated into five machine learning models: Cox regression, LASSO, Decision Tree (DT), Random Survival Forests (RSF), and XGBoost. All models were trained and optimized using 5-fold cross-validation, and the C-index was computed for each model to evaluate its discriminatory performance.

Subsequently, the performance of all models was evaluated in the validation set using time-dependent Receiver Operating Characteristic (ROC) curves, Decision Curve Analysis (DCA), and calibration curves. These methods were used to assess the models’ ability to predict OS and to determine their clinical applicability.

To further explain the models, variable importance plots were generated to visualize the contribution of each predictor in the models. These plots help identify the key features that drive the models’ predictions, providing insight into the relative importance of each variable in predicting survival outcomes.

### Statistical analysis

Categorical variables are presented as counts and percentages, while continuous variables are expressed as either mean ± standard deviation (SD) or median with interquartile range (IQR), depending on the data distribution. Differences between groups were compared using the Chi-square test for categorical variables and Student's t-test or Mann-Whitney U test for continuous variables. The optimal cutoff values for HSP90α and hs-CRP were determined using the maximal survival difference method based on Kaplan-Meier analysis. Survival analysis was carried out with Kaplan-Meier curves and evaluated using the log-rank test. All statistical analyses were conducted using R software, with a significance level set at p < 0.05.

## Result

### Baseline characteristics

A total of 644 patients with advanced HCC were included in the study. The mean age was 55.1 ± 11.2 years, with 63.8% of patients being younger than 60 years. The majority of patients were male (84.5%), and 60.9% had a history of HBV infection. The mean WBC was 6.13 ± 2.85 × 10^9^/L. AFP levels were elevated (≥400 ng/mL) in 49.2% of patients. In terms of liver function, most patients were classified as Child-Pugh A (74.2%), with 26.6% in Child-Pugh B. The majority of patients had multiple tumors (88.0%) and large tumors, with a mean size of 8.75 ± 4.34 cm. PVTT was present in 50.9% of patients, and 69.9% had lymph node involvement. Metastasis was observed in 27.2% of patients, and the majority were classified as BCLC stage C (87.4%), with 12.6% in BCLC stage B.

The optimal cutoff value for HSP90α was 143.3, dividing patients into two groups: HSP90α < 143.3 and HSP90α ≥ 143.3. Significant differences between the two groups were observed in age, HBV, WBC, AFP, Child-Pugh classification, tumor size, PVTT, M status, and BCLC stage.

The optimal cutoff value for CRP was 1.62, dividing patients into two groups: CRP <1.62 and CRP ≥1.62. Significant differences between the two groups were observed in WBC, AFP, ALBI, tumor size, PVTT, N status, and BCLC stage (Table 1).

**TABLE 1:** Baseline characteristics of HCC patients stratified by CRP and HSP90α levels.

Variables	Category	N = 644	CRP <1.62	CRP ≥1.62	P	HSP <143.3	HSP ≥143.3	P
			N = 76	N = 568		N = 359	N = 285	
Age (y)	Mean ± SD	55.1 (11.2)	56.7 (9.78)	54.9 (11.4)	0.135	57.0 (11.2)	52.7 (10.8)	<0.001
	<60	411 (63.8%)	46 (60.5%)	365 (64.3%)	0.611	204 (56.8%)	207 (72.6%)	<0.001
	≥60	233 (36.2%)	30 (39.5%)	203 (35.7%)		155 (43.2%)	78 (27.4%)	
Sex	Female	100 (15.5%)	10 (13.2%)	90 (15.8%)	0.661	62 (17.3%)	38 (13.3%)	0.207
	Male	544 (84.5%)	66 (86.8%)	478 (84.2%)		297 (82.7%)	247 (86.7%)	
HBV	No	252 (39.1%)	34 (44.7%)	218 (38.4%)	0.347	140 (39.0%)	112 (39.3%)	1
	Yes	392 (60.9%)	42 (55.3%)	350 (61.6%)		219 (61.0%)	173 (60.7%)	
WBC (×10^9^/L)	Mean ± SD	6.13 (2.85)	4.73 (1.55)	6.32 (2.94)	<0.001	5.75 (2.56)	6.62 (3.12)	<0.001
NEU	Mean ± SD	8.73 (83.3)	29.5 (232)	5.95 (26.2)	0.379	4.66 (14.7)	13.9 (124)	0.214
AFP (ng/mL)	Mean ± SD	30493 (118333)	8200 (29997)	33476 (125240)	<0.001	8383 (33372)	58344 (170010)	<0.001
	<400	292 (45.3%)	41 (53.9%)	251 (44.2%)	0.317	192 (53.5%)	100 (35.1%)	<0.001
	200–400	35 (5.43%)	3 (3.95%)	32 (5.63%)		24 (6.69%)	11 (3.86%)	
	≥400	317 (49.2%)	32 (42.1%)	285 (50.2%)		143 (39.8%)	174 (61.1%)	
Child–Pugh	A	478 (74.2%)	61 (80.3%)	417 (73.4%)	0.253	287 (79.9%)	191 (67.0%)	<0.001
	B	166 (25.8%)	15 (19.7%)	151 (26.6%)		72 (20.1%)	94 (33.0%)	
ALBI grade	1	171 (26.6%)	36 (47.4%)	135 (23.8%)	<0.001	117 (32.6%)	54 (18.9%)	<0.001
	2	445 (69.1%)	39 (51.3%)	406 (71.5%)		232 (64.6%)	213 (74.7%)	
	3	28 (4.35%)	1 (1.32%)	27 (4.75%)		10 (2.79%)	18 (6.32%)	
Tumor number	1	77 (12.0%)	11 (14.5%)	66 (11.6%)	0.595	49 (13.6%)	28 (9.82%)	0.173
	≥2	567 (88.0%)	65 (85.5%)	502 (88.4%)		310 (86.4%)	257 (90.2%)	
Tumor size (cm)	Mean ± SD	8.75 (4.34)	5.70 (3.65)	9.16 (4.26)	<0.001	7.31 (3.80)	10.6 (4.28)	<0.001
	<3	48 (7.45%)	19 (25.0%)	29 (5.11%)	<0.001	40 (11.1%)	8 (2.81%)	<0.001
	3–5	78 (12.1%)	21 (27.6%)	57 (10.0%)		67 (18.7%)	11 (3.86%)	
	5–10	282 (43.8%)	23 (30.3%)	259 (45.6%)		167 (46.5%)	115 (40.4%)	
	≥10	236 (36.6%)	13 (17.1%)	223 (39.3%)		85 (23.7%)	151 (53.0%)	
PVTT	No	316 (49.1%)	55 (72.4%)	261 (46.0%)	<0.001	218 (60.7%)	98 (34.4%)	<0.001
	Yes	328 (50.9%)	21 (27.6%)	307 (54.0%)		141 (39.3%)	187 (65.6%)	
N status	No	194 (30.1%)	35 (46.1%)	159 (28.0%)	0.002	114 (31.8%)	80 (28.1%)	0.355
	Yes	450 (69.9%)	41 (53.9%)	409 (72.0%)		245 (68.2%)	205 (71.9%)	
M status	No	469 (72.8%)	63 (82.9%)	406 (71.5%)	0.05	287 (79.9%)	182 (63.9%)	<0.001
	Yes	175 (27.2%)	13 (17.1%)	162 (28.5%)		72 (20.1%)	103 (36.1%)	
BCLC stage	B	81 (12.6%)	22 (28.9%)	59 (10.4%)	<0.001	63 (17.5%)	18 (6.32%)	<0.001
	C	563 (87.4%)	54 (71.1%)	509 (89.6%)		296 (82.5%)	267 (93.7%)	

### Biomarker selection

**FIGURE 1:**
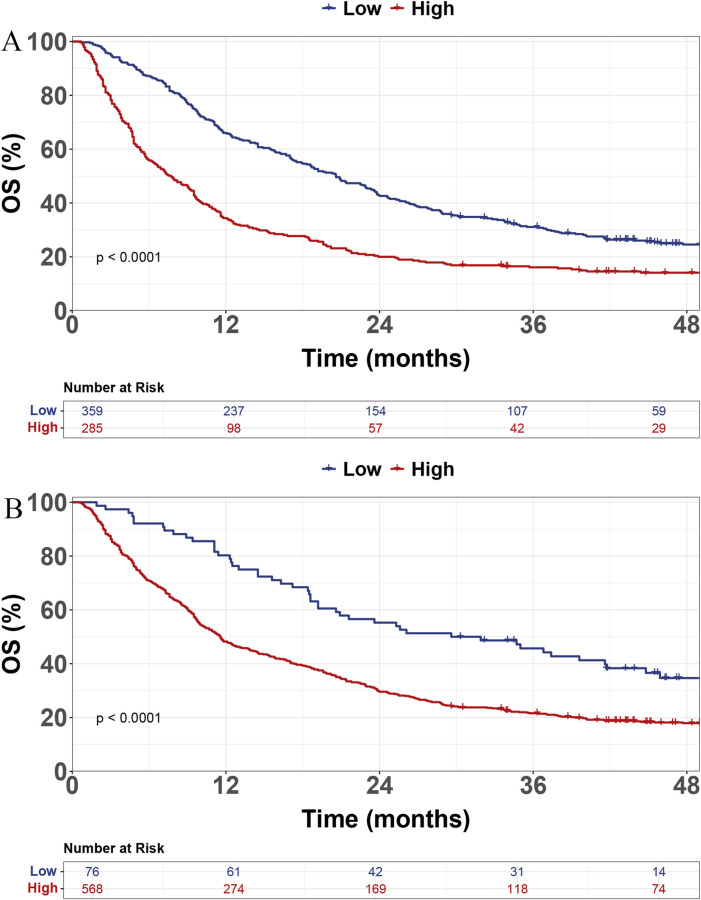
Kaplan-Meier survival curves for overall survival (OS) in advanced hepatocellular carcinoma (HCC) patients based on expression levels of biomarkers. **(A)** High and low expression groups of HSP90α. **(B)** High and low expression groups of hs-CRP.

High expression of HSP90α was associated with significantly shorter OS compared with low expression (7.7 months [95% CI: 6.2–9.5] vs. 20.6 months [95% CI: 17.9–23.6], p < 0.001; Figure 1A). Similarly, high CRP expression was associated with markedly reduced OS compared with low expression (11.6 months [95% CI: 10.2–13.7] vs. 30.8 months [95% CI: 20.6–44.8], p < 0.001; Figure 1B).

**FIGURE 2:**
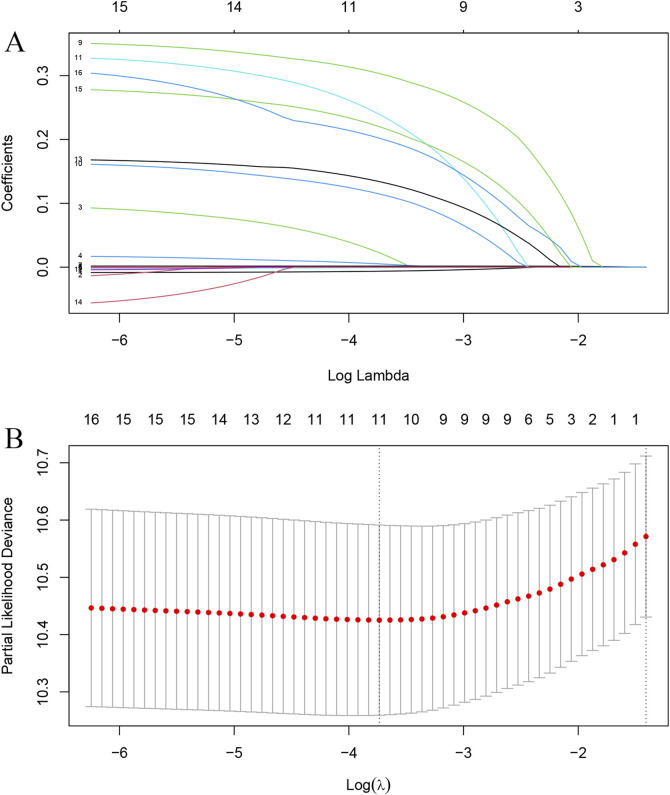
Selection of prognostic factors for overall survival (OS) using LASSO regression **(A)** LASSO coefficient profiles for each variable as a function of the log(lambda) values. The plot illustrates the shrinkage of coefficients as the regularization parameter increases, with the most significant variables remaining after tuning the lambda parameter. **(B)** Partial likelihood deviance for LASSO regression, showing the optimal lambda value selected (indicated by the vertical dotted line) that minimizes deviance while ensuring the inclusion of key prognostic factors.

LASSO regression was used to identify key prognostic factors, including Age, HBV, WBC, CRP, HSP90α, Child-Pugh classification, ALBI, Tumor number, PVTT, M status, and BCLC stage (Figures 2A,B). Subsequently, stepwise Cox regression analysis was performed, and the final independent prognostic factors were determined to be Age, CRP, HSP90α, Child, Tumor number, M status, and PVTT (Table 2).

**TABLE 2:** Univariate and multivariate Cox regression analyses of overall survival in HCC patients.

Variables	Category	HR (Univariable)	HR (Multivariable)	HR (Final)
WBC (×10^9^/L)	Mean ± SD	1.04 (1.01–1.07, p = 0.007)	1.01 (0.98–1.04, p = 0.572)	—
Age (years)	<60	Reference	Reference	Reference
	≥60	0.76 (0.64–0.92, p = 0.004)	0.80 (0.67–0.97, p = 0.022)	0.81 (0.67–0.97, p = 0.025)
HBV	Mean ± SD	1.05 (0.88–1.26, p = 0.559)	—	—
CRP	Low	Reference	Reference	Reference
	High	1.83 (1.37–2.44, p < 0.001)	1.45 (1.07–1.96, p = 0.017)	1.49 (1.10–2.01, p = 0.010)
HSP90	Low	Reference	Reference	Reference
	High	1.90 (1.60–2.25, p < 0.001)	1.50 (1.24–1.81, p < 0.001)	1.55 (1.29–1.86, p < 0.001)
Child–Pugh	A	Reference	Reference	Reference
	B	1.68 (1.38–2.03, p < 0.001)	1.40 (1.13–1.74, p = 0.003)	1.41 (1.13–1.76, p = 0.002)
ALBI grade	1	Reference	Reference	Reference
	2	1.29 (1.05–1.57, p = 0.013)	1.05 (0.85–1.29, p = 0.682)	1.04 (0.84–1.29, p = 0.691)
	3	2.77 (1.81–4.22, p < 0.001)	1.73 (1.07–2.78, p = 0.024)	1.77 (1.10–2.84, p = 0.018)
Tumor number	1	Reference	Reference	Reference
	≥2	1.40 (1.06–1.85, p = 0.017)	1.39 (1.05–1.86, p = 0.023)	1.42 (1.07–1.89, p = 0.015)
PVTT	No	Reference	Reference	Reference
	Yes	1.49 (1.25–1.77, p < 0.001)	1.40 (1.20–1.55, p = 0.027)	1.45 (1.23–1.58, p = 0.023)
M status	No	Reference	Reference	Reference
	Yes	1.52 (1.26–1.84, p < 0.001)	1.29 (1.06–1.57, p = 0.011)	1.28 (1.05–1.55, p = 0.014)
BCLC stage	B	Reference	Reference	Reference
	C	1.73 (1.31–2.28, p < 0.001)	1.25 (0.91–1.70, p = 0.165)	1.34 (1.00–1.80, p = 0.058)

Subsequently, stepwise Cox regression analysis identified Age, CRP, HSP90α, Child–Pugh class, tumor number, M status, and PVTT as the final independent prognostic factors for OS. Specifically, Age ≥60 was associated with reduced risk (HR = 0.81, 95% CI: 0.67–0.97, p = 0.025), while high CRP (HR = 1.49, 95% CI: 1.10–2.01, p = 0.010), high HSP90α (HR = 1.55, 95% CI: 1.29–1.86, p < 0.001), Child–Pugh B (HR = 1.41, 95% CI: 1.13–1.76, p = 0.002), tumor number ≥2 (HR = 1.42, 95% CI: 1.07–1.89, p = 0.015), M status (HR = 1.28, 95% CI: 1.05–1.55, p = 0.014), and PVTT (HR = 1.45, 95% CI: 1.23–1.58, p = 0.023) were all independently associated with worse OS (Table 2).

### HSP90α and CRP

**FIGURE 3:**
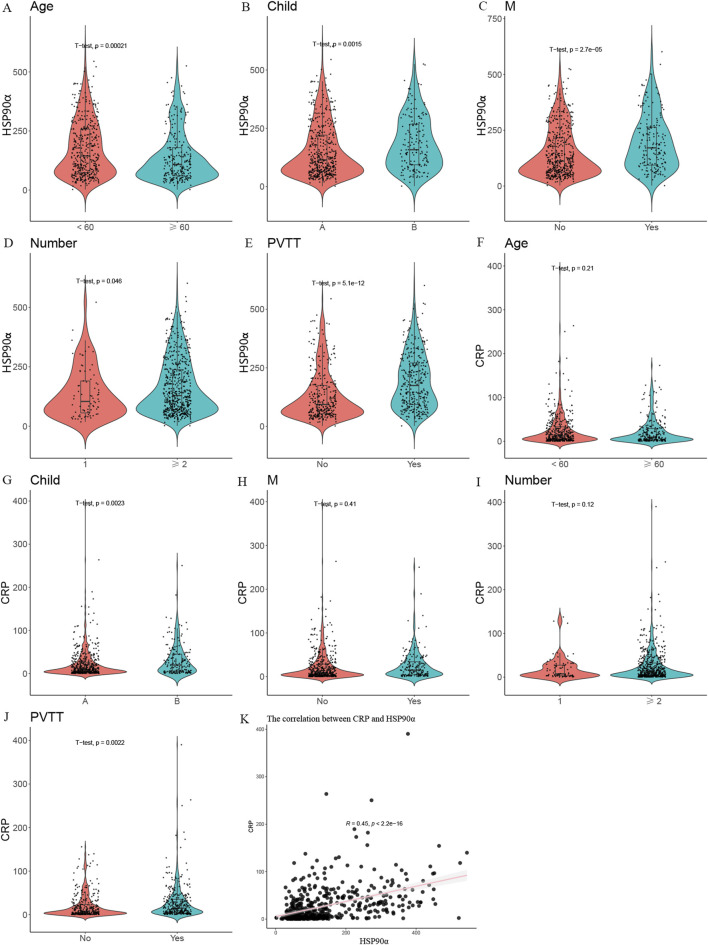
Violin plots and correlation analysis of HSP90α and CRP expression across various clinical and baseline characteristics **(A)** Violin plot showing HSP90α expression in patients <60 vs. ≥ 60 years. **(B)** Violin plot showing HSP90α expression in Child-Pugh A vs. **(B)** **(C)** Violin plot showing HSP90α expression in patients with and without metastasis (M). **(D)** Violin plot showing HSP90α expression in patients with 1 vs. ≥ 2 tumors. **(E)** Violin plot showing HSP90α expression in patients with and without portal vein tumor thrombosis (PVTT). **(F)** Violin plot showing CRP levels in patients <60 vs. ≥ 60 years for CRP levels. **(G)** Violin plot showing CRP levels in Child-Pugh A vs. **(B)** **(H)** Violin plot showing CRP levels in patients with and without metastasis (M). **(I)** Violin plot showing CRP levels in patients with 1 vs. ≥ 2 tumors. **(J)** Violin plot showing CRP levels in patients with and without PVTT. **(K)** Scatter plot showing a positive correlation between CRP and HSP90α (R = 0.45), indicating a significant relationship between these biomarkers.

As shown in Figure 3, significant differences in HSP90α expression were observed across various baseline characteristics. HSP90α levels were significantly higher in patients aged ≥60 years compared to those aged <60 years (p < 0.001, Figure 3A). Higher HSP90α expression was also associated with more advanced disease, including higher Child-Pugh B classification (p = 0.0015, Figure 3B), presence of metastasis (p < 0.001, Figure 3C), greater tumor number (p = 0.048, Figure 3D), and PVTT (p < 0.001, Figure 3E). These findings suggest that HSP90α may serve as a marker of disease progression in HCC.

For CRP, significant differences were observed in Child-Pugh classification (p = 0.0023, Figure 3G) and the presence of PVTT (p = 0.0022, Figure 3J), with higher CRP levels in patients with more advanced liver disease and PVTT. However, no significant differences in CRP were found based on age (p = 0.21, Figure 3F), metastasis (p = 0.41, Figure 3H), or tumor number (p = 0.12, Figure 3I).

Additionally, a significant positive correlation between CRP and HSP90α was observed (R = 0.45, Figure 3K), suggesting a potential interrelationship between these markers in HCC.

### AI model construction

All patients were randomly divided into training and validation sets. In the training set, the independent prognostic factors identified earlier were incorporated into five machine learning models. The C-index for each model were as follows: 0.669 for Cox, 0.647 for LASSO, 0.675 for DT, 0.679 for RSF, and 0.666 for XGBoost. Among these models, RSF demonstrated the highest predictive performance for OS in patients with advanced HCC.

**FIGURE 4:**
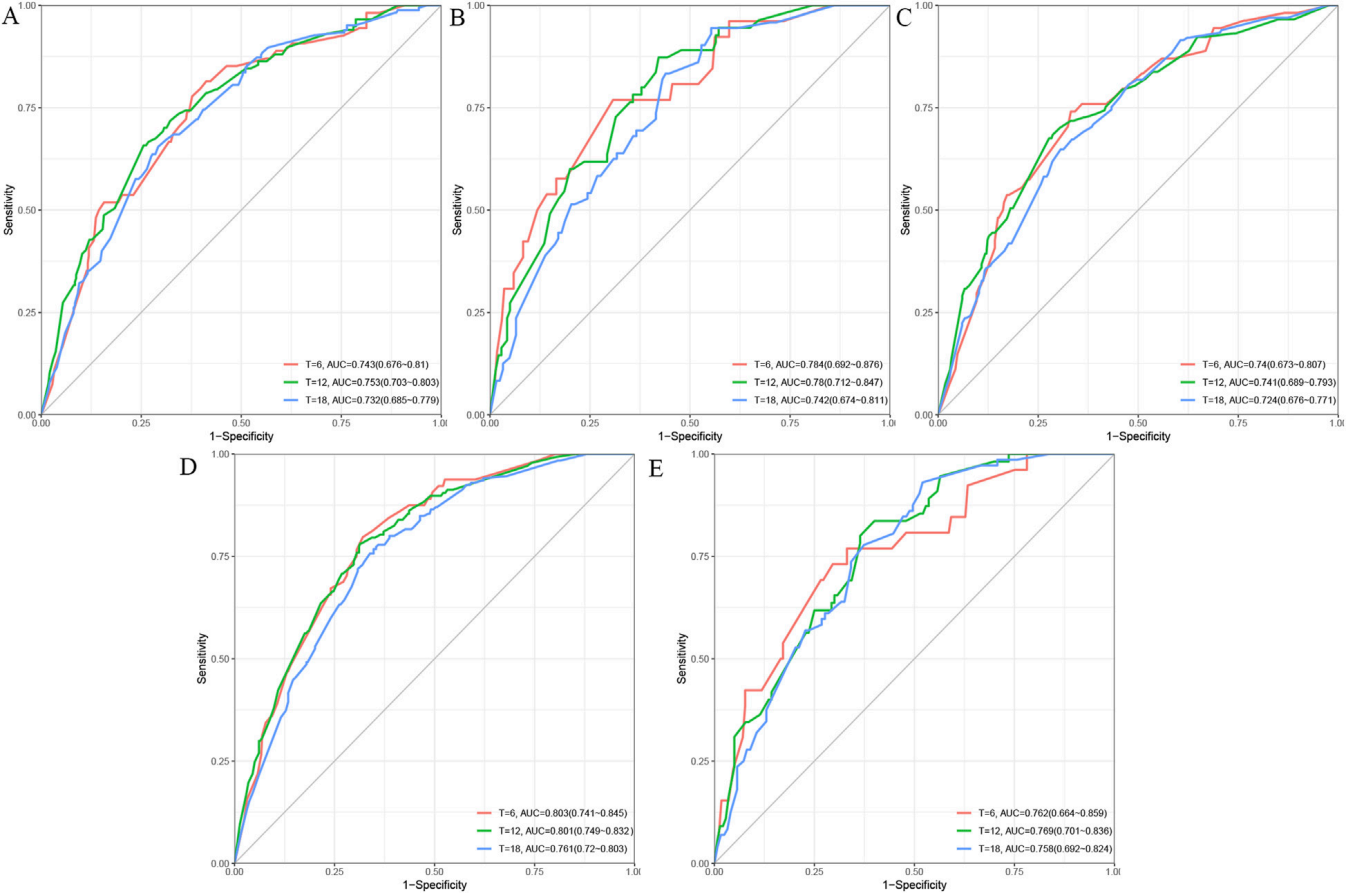
ROC curves were generated for five machine learning models: Cox regression **(A)**, LASSO **(B)**, DT **(C)**, RSF **(D)**, and XGBoost **(E)**.

In the validation set, ROC curves were generated for five machine learning models: Cox regression (Figure 4A), LASSO (Figure 4B), DT (Figure 4C), RSF (Figure 4D), and XGBoost (Figure 4E). The RSF model demonstrated the highest predictive performance, with AUC values of 0.803 at 6 months, 0.801 at 12 months, and 0.761 at 18 months.

### AI model validation

**FIGURE 5:**
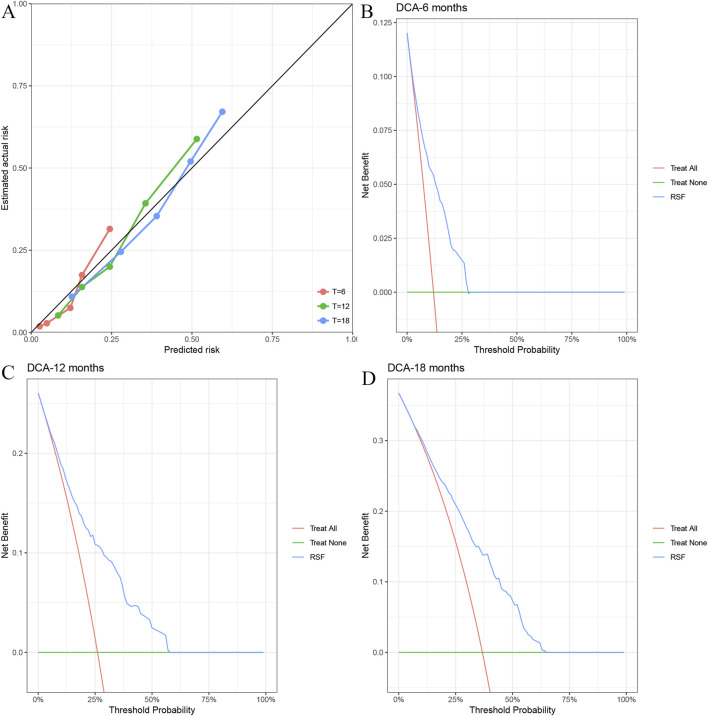
Model performance evaluation using calibration curves and Decision Curve Analysis (DCA). **(A)** Calibration plot for the RSF model at different time points (T = 6, T = 12, T = 18), comparing predicted and actual risk. **(B–D)**: Decision Curve Analysis (DCA) for the RSF model at 6 months **(B)**, 12 months **(C)**, and 18 months **(D)**, comparing the net benefit of the “Treat All,” “Treat None,” and RSF strategies. The RSF model showed the highest net benefit across all time points.

Figure 5A shows the calibration curve for the RSF model, which illustrates the close alignment between the predicted and actual observed risks at various time points. The model demonstrates a good calibration, with the predicted probabilities closely matching the actual survival outcomes for the cohort.

DCA was used to assess the clinical utility of the RSF model at 6 months, 12 months, and 18 months, respectively. The RSF model showed a higher net benefit compared to the “Treat All” and “Treat None” strategies across all time points, indicating its superior predictive value for patient survival. At 6 months (Figure 5B), the RSF model provided the highest net benefit at a threshold probability of approximately 25%. Similarly, at 12 months (Figure 5C) and 18 months (Figure 5D), the RSF model showed superior net benefit compared to the alternative strategies.

**FIGURE 6:**
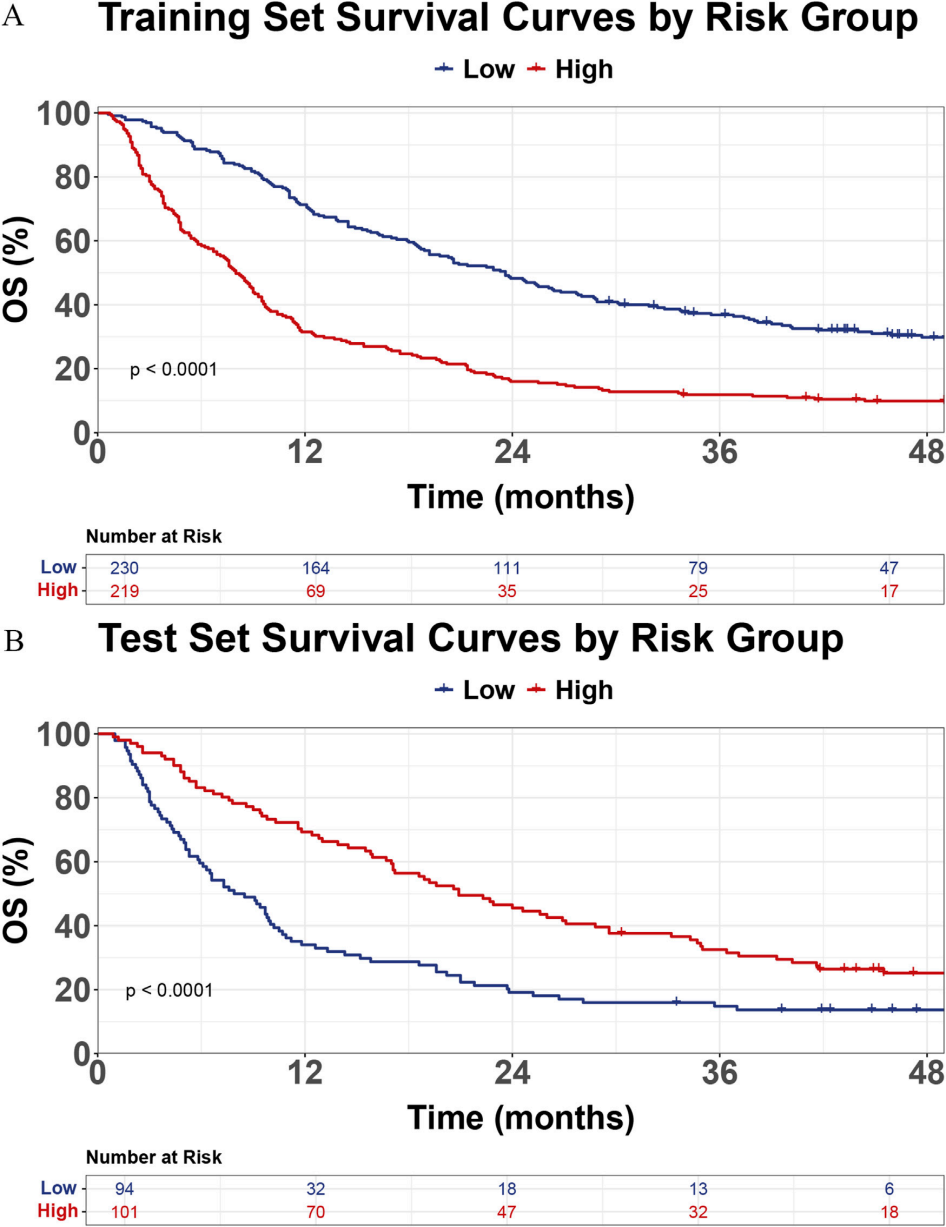
Kaplan-Meier survival curves for overall survival (OS) in training **(A)** and test **(B)** sets based on risk groups.

To further explain the RSF model, a variable importance plot was generated, showing the top five most important features: CRP, Child, M status, HSP90α, and Number of tumors (Supplementary Figure S1). The risk scores for all patients were calculated using the RSF model. In the training set (Figure 6A) and the test set (Figure 6B), the survival curves demonstrate a significant difference in OS between the low-risk and high-risk groups (p < 0.0001). Patients in the high-risk group exhibited a markedly reduced survival probability compared to those in the low-risk group.

## Discussion

HCC remains one of the leading causes of cancer-related deaths worldwide. Most patients are diagnosed at advanced stages, where curative treatments are no longer viable. Despite advances in systemic therapies, particularly ICIs and molecular targeted therapies (Xu K. et al., 2025; Jiang et al., 2025), responses to immunotherapy remain heterogeneous, and reliable biomarkers to predict treatment outcomes are still lacking.

Emerging evidence highlights the active role of stress in modulating tumor immunity (Li W. et al., 2025). Chronic stress, sympathetic nervous activation, and stress-induced inflammation can influence immune responses, impacting both tumor progression and therapeutic efficacy (Noverati et al., 2022; Zhang et al., 2020). Circulating biomarkers such as HSP90α and hs-CRP offer insight into tumor immunity. HSP90α, a stress-inducible molecular chaperone, stabilizes several oncogenic and immune checkpoint-related proteins, while hs-CRP, an IL-6–driven acute-phase reactant, reflects systemic inflammation under neuroendocrine influence (Deng et al., 2025; Chen et al., 2024).

Understanding how this neuroimmune stress–inflammation axis interacts with immune modulation could enhance treatment decisions for advanced HCC. To address the complexities of the interplay between stress, inflammation, and immune responses, this study incorporated machine learning models to assess the prognostic value of HSP90α and hs-CRP in patients receiving immunotherapy.

Our findings demonstrate the significant prognostic value of both HSP90α and hs-CRP in predicting OS in advanced HCC. High expression of HSP90α was significantly associated with shorter OS, confirming its role as a marker of disease progression. Elevated HSP90α levels contribute to immune escape by stabilizing oncogenic proteins, which can impair the effectiveness of immunotherapies. Our study showed that patients with high HSP90α expression had significantly shorter OS (7.7 vs. 20.6 months, p < 0.001). These results suggest that HSP90α could be a reliable biomarker for predicting immunotherapy response and assessing disease severity.

Similarly, high hs-CRP expression was associated with worse survival outcomes, supporting the role of inflammation in tumor progression and immune suppression. Elevated CRP levels reflect systemic inflammation driven by neuroendocrine factors, which are known to contribute to an immunosuppressive tumor microenvironment (Du et al., 2024). In our study, patients with high CRP levels had significantly shorter OS (11.6 vs. 30.8 months, p < 0.001). This highlights the value of hs-CRP as a prognostic marker in HCC, suggesting that targeting inflammation could improve therapeutic responses.

To further improve the predictive accuracy of these biomarkers, we employed machine learning models. By integrating clinical and biological data, we constructed a robust prognostic model using Cox regression, LASSO, DT, RSF, and XGBoost. The RSF model demonstrated the highest predictive performance, with AUC values of 0.803 at 6 months, 0.801 at 12 months, and 0.761 at 18 months, outperforming the other models. The RSF model’s superior performance underscores the advantage of machine learning in capturing complex, nonlinear interactions between clinical and molecular factors, improving predictions of OS in advanced HCC (Hsieh et al., 2023; Xia et al., 2024; Zhao et al., 2024; Saillard et al., 2020; Song et al., 2025; Zhao et al., 2025).

The clinical utility of the RSF model was validated through calibration curves and DCA, which demonstrated that the RSF model offered the highest net benefit compared to other strategies at all time points (6, 12, and 18 months). This suggests that the RSF model could be a valuable tool for guiding treatment decisions, helping clinicians tailor therapy based on the patient’s risk score. The variable importance plot identified key features influencing survival predictions, including CRP, Child-Pugh classification, M status, HSP90α, and tumor number.

Despite these promising results, our study has several limitations. As a retrospective analysis, selection bias may have influenced the findings. In addition, the specific types of PD-1 inhibitors used were not completely uniform across patients, and this heterogeneity may have introduced variability in treatment response. Prospective validation of the models and biomarkers in independent cohorts is necessary to confirm the robustness of our conclusions. Although HSP90α and hs-CRP showed strong associations with OS, the mechanistic interaction between these biomarkers within the HCC immune microenvironment remains insufficiently understood. Future basic and mechanistic studies are planned to further validate and elucidate the pathways through which HSP90α and hs-CRP jointly influence immune regulation, immune evasion, and tumor progression. Moreover, the application of machine-learning models in clinical settings requires careful consideration of interpretability and integration into routine practice.

## Conclusion

In conclusion, this study highlights the prognostic significance of HSP90α and hs-CRP in advanced HCC. The RSF model demonstrated superior predictive performance, offering a promising tool for personalized treatment strategies. The integration of biological markers with machine learning models could enhance the precision of treatment decisions and improve patient outcomes in advanced HCC.

## Data Availability

The original contributions presented in the study are included in the article/Supplementary Material, further inquiries can be directed to the corresponding authors.
