# Supplementary figures and images for: Exploring HSP90α and hs-CRP using AI models to predict prognosis in advanced hepatocellular carcinoma treated with PD-1 inhibitors and targeted therapy
Source: Front Pharmacol. 2025 Dec 12;16:1726967. doi: 10.3389/fphar.2025.1726967 (PMC12741144; doi:10.3389/fphar.2025.1726967)

**Supplementary Fig. 1**. Variable importance plot for the RSF model.


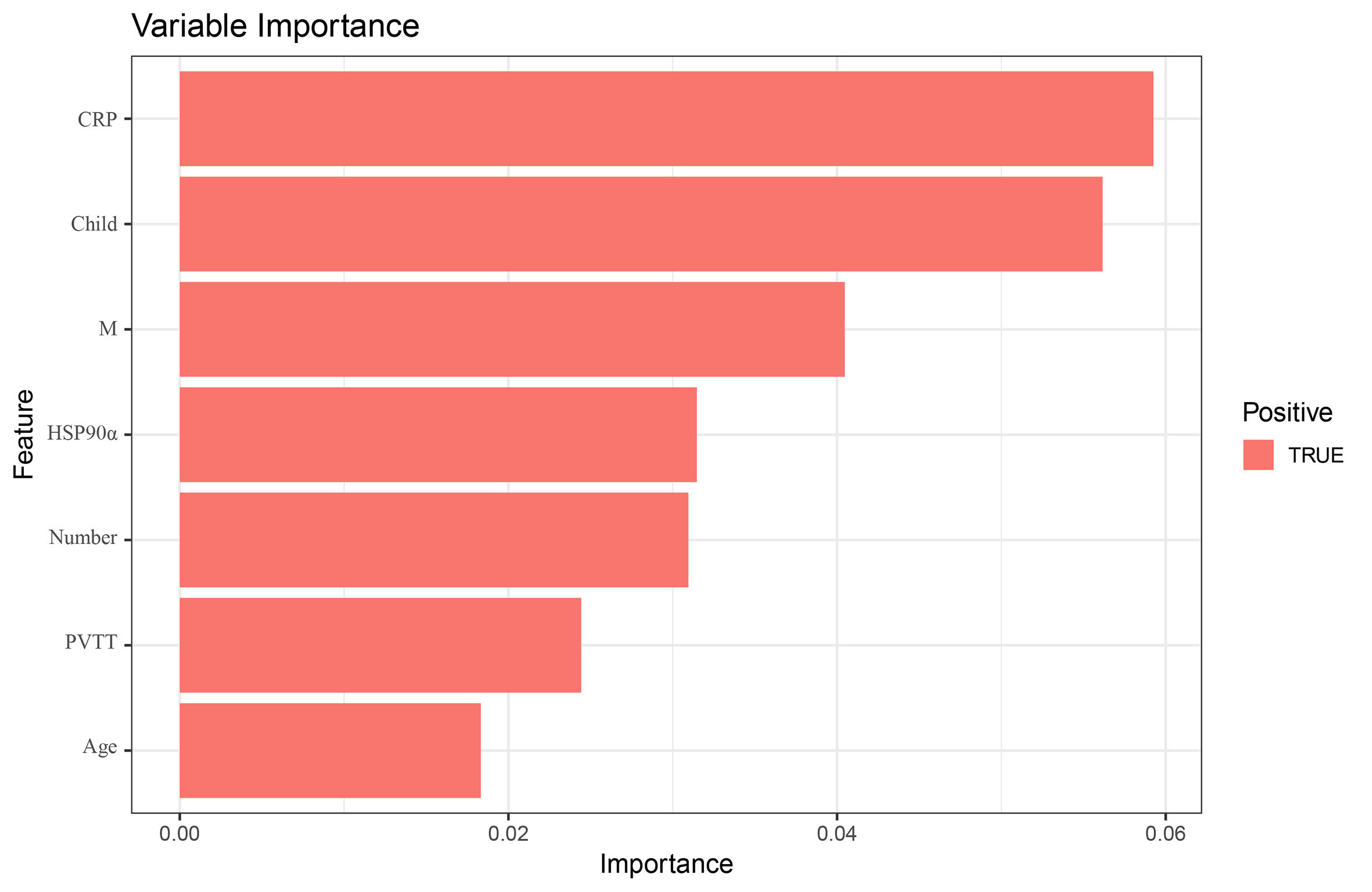

Supplement: Supplementary file 1 [file Supplementaryfile1.docx]
